# Some Laws of Health

**Published:** 1919-10-04

**Authors:** 


### SOME LAWS OF HEALTH.

For some time it had been generally recognised that there was an enormous proportion of physical unfitness amongst the population of these Islands. Public health and other medical and municipal authorities had been persevering for many years in their endeavours to eradicate, to an ever-increasing extent, the causes which led to disease amongst our citizens. But it was not until the present war, under conditions which called for mobilisation of the entire Country, that these facts were generally recognised by the masses, who, like the proverbial ostrich, buried their thinking selves in the sands of the desert of Ignorance. Ignorance has always retarded progress, and, if for no other reason, the war will have proved to be a blessing in disguise if it has brought the country to a realisation of its great responsibilities in providing a healthier race.

### The Adaptation of the Principles of Health.

To deal with the question in a scientific, and, therefore, the only rational, manner, we must start at the beginning and examine carefully the conditions under which the foundations of new lives are laid down.

It is, theoretically, an ideal law that the love between two of the opposite sex should find its fulfilment and consummation in the production of a third, but we must never forget that the parents are under a heavy responsibility when they invite Nature to fashion another human being out of the results of their union. It should be compulsory on every man and woman that, before mating, they should seek the advice of a medical man, and the law should make it a crime for people knowingly to produce children when one or both is suffering from uncured syphilis, tuberculosis, carcinoma, insanity, uncontrolled drinking, or drug-taking.

During pregnancy the future mother should receive special care and attention. She should, as far as possible, be prevented from any excess, physical or mental, for she is perhaps the greatest asset that the nation possesses, and should always be regarded as such.

The rearing of the new-born infant has been sadly misdirected by many, but, as statistics show, the great numbers of deaths from summer diarrhœa, etc., have already been largely minimised by a more strict attention to Nature's laws, the natural outcome of a better understanding of them. It is the bounden duty of every mother to provide, to the utmost of her ability, the natural nourishment for her child; in so doing she is not only benefiting the latter but also herself. Modern conditions may possibly have lessened the number of those able to suckle their offspring; but there are also many who selfishly decline to use what Nature has so ably provided for them.

### Adolescence.

The period of adolescence demands no less care, and the treatment may be summed up as follows: Sensible nourishment, healthy exercise, and an education in the enjoyment of Nature's great gifts without abuse. There have been many complaints during the last twelve months of parents who found that their children were not receiving adequate nourishment whilst at school, and this though extra fees were charged to meet the increased cost of living; this must never be allowed, for wherever growth of tissues is taking place there is an increased necessity for fuel of the best quality.

In choosing an occupation, it is essential to bear in mind the natural bent of the individual which has shown itself during the previous years. Many a young life has been spoilt by choosing a totally unsuitable means of livelihood. From early manhood to the onset of old age the citizen should be able to give to his country the best output, increasing as his experience becomes greater. But the maximum value of such a complicated machine as the human body can only be obtained under conditions of sound health. "*Mens sana in sano corpore*" is as true to-day as when it was first taught by the Roman sages.

The value of efficient nasal respiration is becoming more and more evident as our knowledge increases. To the condition of diseased tonsils and adenoids, with deflected nasal septum and hypertrophic rhinitis, so often the results of the former, may, in many cases, be ascribed the blame for the lack of efficiency. Again, tubercle bacilli, ever ready to seek a home in weakened tissues, find a fruitful nidus in diseased tonsils and adenoids, etc. A great deal has been done already by the medical inspection and treatment of children in L.C.C. and other schools, and until the masses have learnt to appreciate even the rudimentary laws of health some form of compulsory medical supervision will be necessary.

### The Ministry of Health.

There is now a golden opportunity for the newly created Ministry of Health, which should sweep away many of the old shibboleths with the broom of national reform. We have, and always shall have, much destructive criticism; let us now harness ourselves to constructive organisation, and, instead of blindly groping in the slough of superstitious ignorance, let us tackle this great and vital problem on common-sense lines. Cleanliness in mind and body should be engendered in every young mind; marriages should be encouraged amongst all classes at an age when the healthiest offspring can best be produced, and the health-giving delights of an agricultural life should be brought home to the minds of thousands who at the present time drift into our big cities, to be sooner or later swallowed up into the vortex of degeneration.

